# Biomimetic Ghost Nanomedicine-Based Optotheranostics for Cancer

**DOI:** 10.1021/acs.nanolett.4c01534

**Published:** 2024-06-07

**Authors:** Rajendra Prasad, Vaskuri G. S. Jyothi, Nagavendra Kommineni, Ravi Teja Bulusu, Bárbara B. Mendes, Jonathan F. Lovell, João Conde

**Affiliations:** † School of Biochemical Engineering, Indian Institute of Technology (BHU), Varanasi, Uttar Pradesh 221005, India; ‡ Department of Pharmaceutical Sciences, University of Tennessee Health Science Center (UTHSC), Memphis, Tennessee 38163, United States; § Center for Biomedical Research, Population Council, New York, New York 10065, United States; ∥ Department of Pharmaceutical Sciences, Florida A&M University, Tallahassee, Florida 32307, United States; ⊥ NOVA Medical School|Faculdade de Ciências Médicas, NMS|FCM, Universidade NOVA de Lisboa, Lisbon 1169-056, Portugal; # ToxOmics, NOVA Medical School|Faculdade de Ciências Médicas, NMS|FCM, Universidade NOVA de Lisboa, Lisbon, 1169-056, Portugal; ∇ Department of Biomedical Engineering, University at Buffalo, State University of New York, Buffalo, New York 14260, United States

**Keywords:** Biomimetics, Cell Ghosts, Optotheranostics, Phototherapeutics, Solid tumors

## Abstract

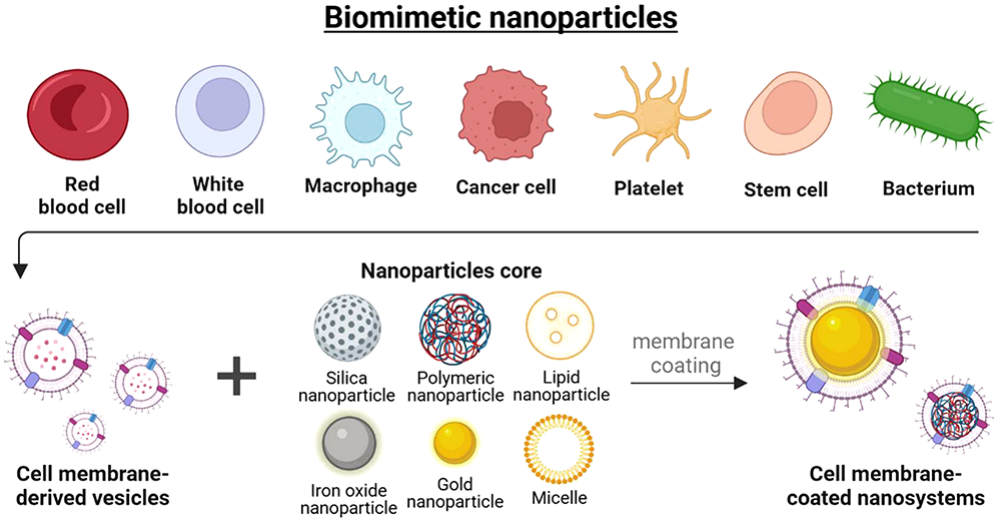

Theranostic medicine combines diagnostics and therapeutics, focusing on solid tumors at minimal doses. Optically activated photosensitizers are significant examples owing to their photophysical and chemical properties. Several optotheranostics have been tested that convert light to imaging signals, therapeutic radicals, and heat. Upon light exposure, conjugated photosensitizers kill tumor cells by producing reactive oxygen species and heat or by releasing cancer antigens. Despite clinical trials, these molecularly conjugated photosensitizers require protection from their surroundings and a localized direction for site-specific delivery during blood circulation. Therefore, cell membrane biomimetic ghosts have been proposed for precise and safe delivery of these optically active large molecules, which are clinically relevant because of their biocompatibility, long circulation time, bypass of immune cell recognition, and targeting ability. This review focuses on the role of biomimetic nanoparticles in the treatment and diagnosis of tumors through light-mediated diagnostics and therapy, providing insights into their preclinical and clinical status.

Light, a form of electromagnetic radiation, exhibits both particle- and wave-like characteristics. Electromagnetic waves have specific properties, including wavelength (λ, the distance between successive peaks), frequency (number of oscillations per second), and amplitude (the difference between the trough and peak). Within electromagnetic radiation, energy particles, called photons, move at a constant speed of 3 × 10^8^ m/s. Consequently, a combination of waves comprises photons traveling with varied amplitudes and frequencies, scattering, and absorption. These phenomena are reflected in various objects, including biological materials.^1^ As research has deepened our understanding of how to harness this energy, applications of electromagnetic radiation in medical therapy have advanced significantly worldwide. Laser therapy, for instance, has become a common treatment in certain medical specialties and has been proven to be effective for numerous chronic diseases without causing adverse side effects.

Various forms of radiation therapy have been utilized over the past four decades as therapeutic interventions. Radiation therapy involves the targeted application of specific wavelengths of light to tissues to promote healing and functional recovery.^2^ Owing to its properties, near-infrared (NIR) light has emerged as a promising therapeutic modality for the diagnosis of diabetes, epilepsy, metabolic myopathy, and cardiac diseases, as well as for the treatment of acute and chronic musculoskeletal injuries and various cancers. NIR light modalities emit photons within a specific narrow bandwidth with wavelengths ranging from 700 to 1000 nm. Hence, the NIR region is often termed as the “therapeutic window”. Examples of NIR light modalities include class 3 and class 4 lasers, as well as light-emitting diodes (LEDs). LEDs emit light in the red-to-infrared range with intensities within the class 3 laser range.^3^ The NIR spectrum results from the absorption of atomic groups of CH, NH, and OH containing hydrogen atoms, leading to overtones and stretching and bending vibrations. Analysis of the NIR spectrum provides an understanding of changes in the body.^4^ Recently, NIR has gained attention in the field of oncology for diagnosis and treatment, which is termed phototherapy. With advances in the field of NIR, nanotechnology has been integrated with phototherapy, resulting in better outcomes in the field of oncology.

Incorporating advancements in biomimetic strategies, the convergence of nanotechnology and photomedicine represents a significant improvement. The design of biomimetic nanoparticles, modeled after biological systems, enhances the specificity and efficacy of the NIR treatment. These NPs can mimic natural biological processes, allowing homologous targeting and evasion of the immune system, resulting in increased accumulation at tumor sites and prolonged systemic circulation. The integration of biomimetics with phototherapeutic potential is a powerful and precise approach to oncological treatments. It allows for the selective destruction of cancerous cells while sparing healthy tissue, targeted delivery of therapeutics, and the potential to activate the body’s immune response against tumors, creating more effective, less invasive, and highly personalized cancer treatment modalities attuned to the complex dynamics of the human body.

## How Do Biomimetic Nanoparticles Enhance Therapeutic Strategies in Cancer Nanomedicine?

Cancer is one of the most lethal diseases worldwide. Chemotherapy was introduced for the treatment of cancer; however, it is associated with numerous side effects. To address this issue, a nanotechnology strategy has been implemented for the targeted delivery of drugs to the site of action, minimizing the associated side effects. With the approval of Doxil by the US FDA, more research has focused on the use of nanotechnology in the delivery of chemotherapeutics.^5^

**Figure 1:**
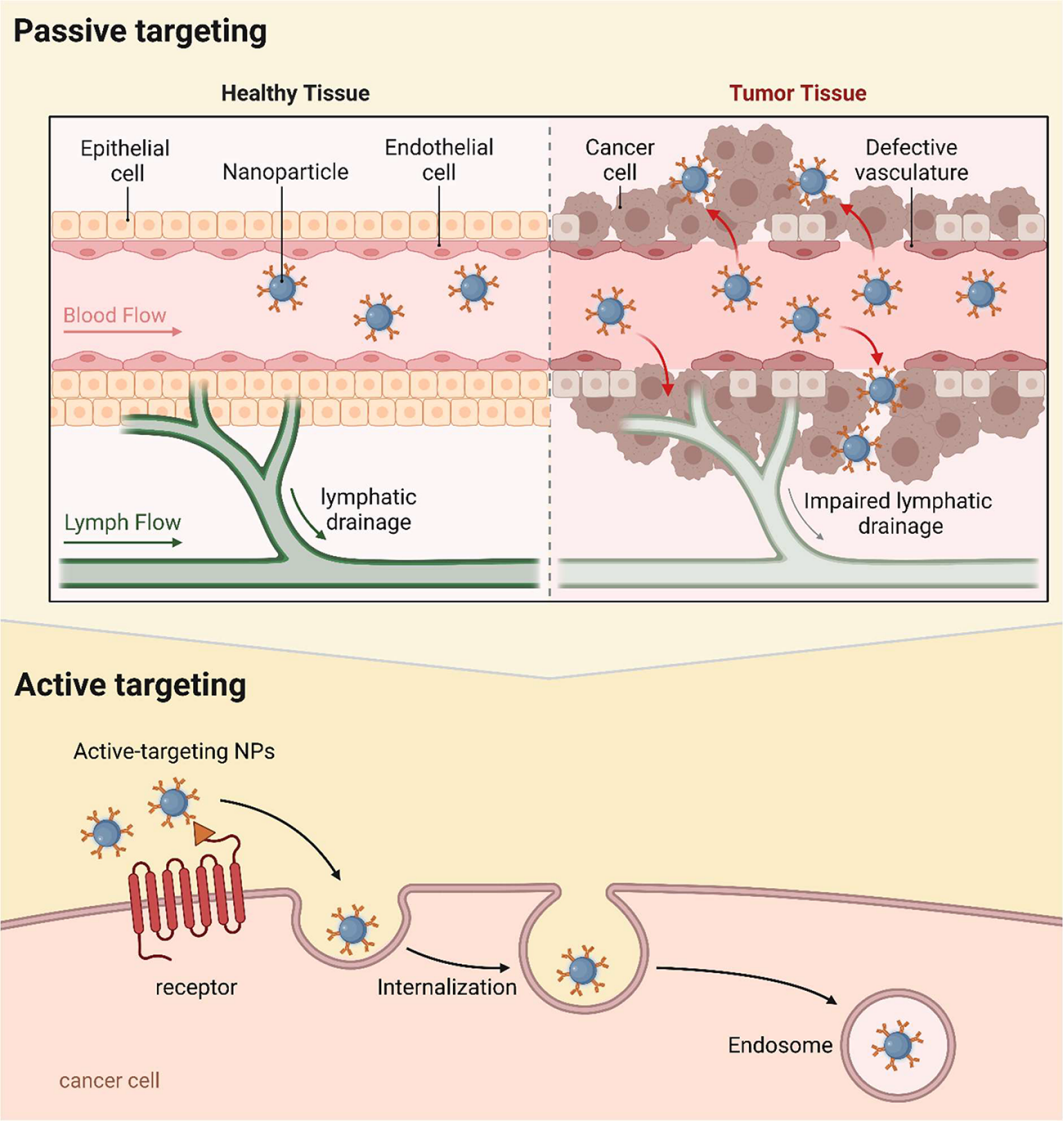
Passive versus active nanoparticle targeting in cancer therapy. Mechanisms of passive and active targeting in NP-mediated drug delivery systems for cancer treatment, showing how NPs circulate through healthy tissue versus tumor tissue and the specific binding and internalization in cancer cells through active targeting. Adapted from ref (8). Available under CC-BY 4.0. Copyright 2022 MDPI, Basel, Switzerland.

The incorporation of nanotechnology in cancer treatment has alleviated side effects by ensuring passive or active targeting, offering precise delivery to the site of action, minimizing toxicity, and enhancing permeability, thereby increasing the potency of therapy. Tumor cells also favor the accumulation of nanoparticles (NPs) in tumor tissues through the phenomenon called the Enhanced Permeation and Retention (EPR) effect.^6^ The EPR effect is due to enhanced neovascularization of the tumor resulting from high proliferation and imperfect angiogenesis with large pores in the walls of newly formed vessels, resulting in passive accumulation and retention of NPs in the tumor tissue (Figure 1). Moreover, passive targeting does not distinguish between normal and diseased cells and may lead to off-target side effects. It also depends on the physiological condition of the individual. This leads to inconsistency in the delivery of cancer therapeutics to the site of action. As passive targeting relies on the EPR effect, deep penetration of the therapeutics into the tumor tissues cannot be anticipated. Active targeting is used for the selective accumulation of NPs in tumor tissues, where a targeting ligand is tagged to the surface of the NPs, resulting in the active accumulation of drugs entrapped in the tumor tissue. Despite the numerous advantages of targeting NPs to the tumor site, there are more bottlenecks to overcome for efficient delivery. It should be noted that ligand-based active targeting involves complexity in tagging the ligand to the nanoparticles coupled with cost of synthesis. The expression of target receptors on the surface of cancer cells varies, which can reduce the delivery efficiency. The ligands fabricated on the surface of nanoparticles can trigger an immune response, thus hampering the advantage of active targeting.

Numerous types of nanocarriers have been explored for cancer treatment, leading to promising results. Polymeric, lipid-based, protein-based, cell-derived biomimetic, and vesicular-type nanocarriers have been used as delivery systems for cancer cells.^7^ In particular, biomimetic nanoparticles are biological in nature and can be loaded with various bioactive imaging and therapeutic probes. Most importantly, for localized tumor targeting followed by long-distance communication, these nanoparticles require transport through blood circulation to reach the target sites. However, the mechanisms underlying the tumor entry–exit of biomimetic nanoparticles are not yet well understood. Interestingly, systemically administered biomimetic nanoparticles demonstrate specific biodistribution and site-selective tumor targeting. However, they still face various biological barriers during blood circulation. Biomimetic nanoparticles have the advantages of both passive targeting via the EPR effect and active targeting by taking advantage of functionalized biomimetic membranes. The presence of biomimetic membranes or extracellular matrix components on the surface also plays a pivotal role in enhancing the penetration ability of NPs and reducing the immune response, making them more biocompatible. Extravesicular and biomimetic nanocarriers have also been implemented as novel technologies.

Despite the advancements in nanotechnology in chemotherapy, the results still lead to numerous side effects, and the focus is shifting to the amalgamation of phototherapy with nanotechnology.

NPs that absorb NIR light are being studied in the medical field, particularly for the treatment of cancer as phototherapy.^9^ Metallic NPs, particularly gold and silver NPs, have been extensively studied for therapeutic applications. They absorb light efficiently and convert it into heat, leading to localized hyperthermia and tumor destruction. Several gold-based nanomaterials, such as gold nanorods and gold nanoshells, are in the preclinical stages of various cancers and have demonstrated promising results in animal studies.^10,11^ Nonmetallic NPs, such as carbon-based nanomaterials (e.g., carbon nanotubes and graphene), have unique photothermal properties and can be functionalized for targeted therapies. Carbon-based NPs have been investigated in preclinical studies for their phototherapeutic potential. They showed promising results in enhancing the thermal ablation of tumors when combined with laser irradiation.^12^ Biological photonanomedicine involves the use of light-responsive biological agents, such as photosensitizers or genetically engineered cells (biomimetics), for targeted therapies. Photosensitizers generate reactive oxygen species (ROS) upon light activation, leading to cell damage. Photosensitizer-based therapies, such as photodynamic treatment (PDT), are in both clinical and preclinical stages. Photosensitizers are used for various cancers, including skin, lung, and esophageal cancers, and have been actively studied for their efficacy and safety.^13^

## Why Do Optically Active Biomimetic Nanoparticles Outperform Other Stimuli-Responsive Nanoparticles?

NPs are well versed in cancer treatment; however, stimuli-responsive nanoparticles are employed for target-specific drug delivery. These stimuli-responsive nanoparticles are designed in such a way that they are sensitive to cancer pathology, which aids in specifically targeting cancerous cells. Cancer-specific stimuli include pH, temperature, enzymes, and redox microenvironment.^14^ Additionally, external stimuli, such as heat, light, magnetic fields, and ultrasound, also contribute to stimuli-responsive nanoparticles. Of all stimuli-responsive nanoparticles, pH-responsive nanoparticles have been well studied in cancer drug delivery. The pH of cancerous tissue is more acidic (pH 4.5–5.5) than that of normal tissue, exhibiting a pH of 5.7–7.8.^15^ This acidic pH is due to the high rate of glycolysis in cancerous cells. pH-responsive nanoparticles are designed in such a way that an ionizable chemical group is introduced into the structure of nanoparticles such as amines, carboxylic acids, and phosphoric acids, where the acidic pH in the cancer tissue leads to either accepting or donating protons, thereby changing the physicochemical properties of the nanomaterial and triggering the release of the drug. The other strategy involves the formation of acid-labile chemical bonds, where the molecules degrade in the acidic environment of cancer tissue, leading to the release of the drug. Redox-responsive nanoparticles have also been widely explored as stimuli-responsive nanoparticles. In these nanoparticles, the difference in the redox potentials of normal and cancer cells is taken as an advantage for the delivery of drugs by incorporation of oxidation- or reduction-sensitive chemical groups in the nanoparticles. Disulfide bonds are widely used for constructing reduction-responsive nanocarriers, which are susceptible to quick breakage by glutathione tripeptide (γ-glutamyl-cysteinyl-glycine, GSH) via a dithiol–disulfide exchange mechanism in cancer tissue.^16^ Enzyme-responsive nanocarriers have been designed for targeted delivery in cancer tissues, triggering the overexpression of oxidoreductase and hydrolase enzymes in cancer physiology. With these advances in stimuli-responsive nanoparticles, most nanoparticles face hurdles in terms of safety and efficacy because they are recognized as foreign objects by physiological systems and are limited by biological barriers, including immune clearance and opsonization.

Different approaches have been developed for the accurate treatment of cancer by manipulating the physicochemical properties of nanoparticles by stealthy synthesis of the surface or by functionalization. More recently, biomimicking cells have been the focus of cancer treatment, where nanoparticles imitate the physiological characteristics of a living cell. Biomimetic materials are embedded or coated onto their surfaces to replicate the biological characteristics and functions of native cells.^17^ This permits biomimetic nanoparticles to escape from immune clearance, enabling biocompatibility, targetability, and retention for extended periods of time. Various biomaterials are used for biomimicking nanoparticles, including erythrocytes, neutrophils, and cancerous cell-derived membranes. The incorporation of optically active molecules into biomimetic nanoparticles leads to therapeutic and diagnostic effects in cancer, aligning with the advantages of biomimetic nanoparticles.^18^

Optically active molecules have attracted the attention of the scientific fraternity for the treatment of cancer. Optically active molecules are entrapped in lipid nanoparticles, which aids in cancer phototherapy. Lipidic nanoparticles assist in the delivery of photoactive compounds to tumorous tissue.^19^ However, current research presents biomimetic functional materials made from bacterial outer membrane vesicles, extracellular vesicles, and cell membranes in recognition of the shortcomings of these approaches.^20,21^ Because these materials can activate antitumor immunity, increase drug targeting, and evade the immune system, they have great potential for treating tumors.^22^

**Figure 2:**
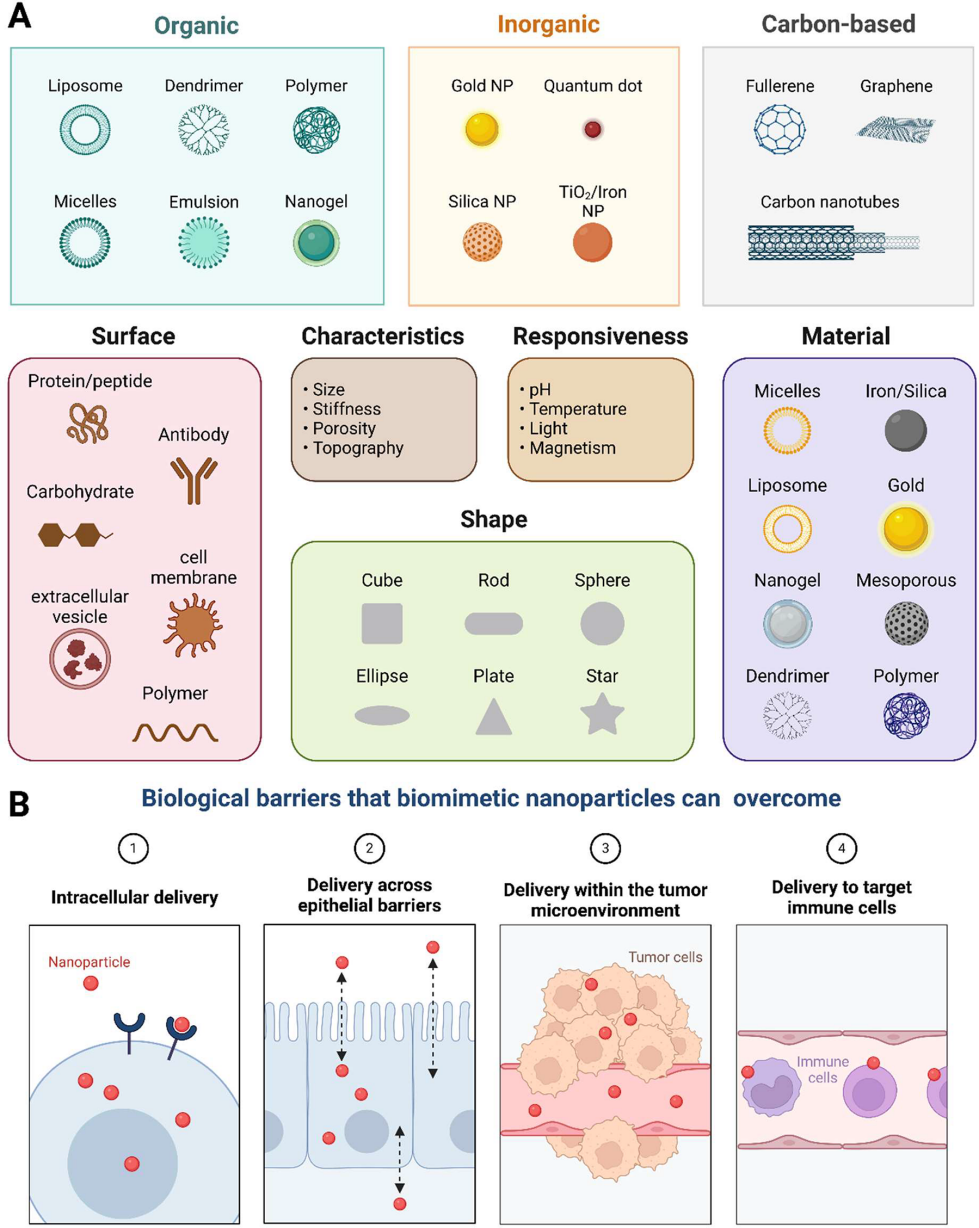
Optically active biomimetic nanoparticles versus stimuli-responsive nanoparticles. (A) Classification and characteristics of nanoparticles, categorizing nanoparticles into organic, inorganic, and carbon-based types, each with unique structures, such as liposomes and dendrimers, highlighting key nanoparticle characteristics such as size and responsiveness, which are crucial for various applications in nanomedicine. Various types of biomimetic nanoparticles have been developed, including cell-membrane-coated, targeting ligands, and natural protein-based nanoparticles. (B) Advantages and applications of biomimetic NPs. Advancements in hybrid cell-derived biomimetic materials in overcoming biological barriers include (1) facilitating intracellular delivery, (2) crossing epithelial barriers, (3) navigating the tumor microenvironment, and (4) targeting immune cells, thereby highlighting their therapeutic potential in drug delivery and cancer treatment. Owing to the shortcomings of the existing tumor treatment approaches, phototherapy has emerged as a promising alternative. Without the need for drugs, phototherapy, which transforms light energy into chemical or thermal energy, offers a more straightforward and potent approach to tumor treatment. Both photothermal therapy (PTT) and PDT have been investigated in the context of phototherapy. Designed by Biorender.

Artificial bionic membranes, known as liposomes, have a high drug-loading capacity and are easily modifiable, making them promising options for drug administration. However, an inherent drawback of liposomes is that they are not naturally capable of active targeting. To achieve this, scientists have investigated hybridization options such as combining lipids with bacterial outer membrane vesicles, extracellular vesicles, or cell membranes.^23^ The resulting lipid-hybrid cell-derived biomimetic functional materials seek to overcome the shortcomings of current biomimetic materials, such as their low drug-loading capacity and complicated fabrication processes, by combining the benefits of liposomes and cell-derived components. Polymers, nanomaterials, and liposomes have been integrated with bacterial outer membrane vesicles and tumor-derived extracellular vesicles in this field of study (Figure 2).^24^

Liposomes, for example, have emerged as key players in the development of lipid-hybrid cell-derived biomimetic functional materials owing to their bilayer structure, which is similar to that of biomembranes. Their high drug-loading capacity, inherent biocompatibility, and modifiability make them adaptable carriers that can encapsulate therapeutics that are hydrophilic or hydrophobic.^23^ Liposomes are promising candidates for cancer therapies.^25^ In one study, mesoporous manganese dioxide (H-MnO_2_) was combined with collagenase (Col) wrapped on the surface and doxorubicin was loaded into the core to create a liposomal system. H-MnO_2_-Dox-Col NPs were coated with a pH-sensitive liposome and an inflammation-targeted RAW264.7-cell membrane to generate MP@H-MnO_2_-Dox-Col, a biomimetic membrane (MP) that improved the therapeutic efficacy of the compound. The multifunctional nature of this biomimetic nanodelivery system was demonstrated through both in vitro and in vivo experiments. Owing to its efficient penetration into tumor tissue, reduction of hypoxia in the tumor microenvironment (TME), pH-sensitive drug release, and targeted distribution of Dox, the results demonstrated its capacity to maximize the efficacy of Dox while limiting cardiotoxicity. Combining this biomimetic nanosystem with first-line clinical therapy holds promise for future breast cancer interventions. This biomimetic nanosystem demonstrated promising antitumor activity, establishing it as a possible therapeutic agent for breast cancer treatment.^26^ Another example is the integration of the capabilities of ultrasmall platinum nanoparticles (nano-Pt) and verteporfin (VP) to create a synergistic effect through combined chemotherapy and PDT. During the synthesis, folic acid was used as the stabilizing agent in a one-step reduction process, resulting in nano-Pt, which has a diameter of 3–5 nm. Using the reverse-phase evaporation method, these nano-Pt nanoparticles were encapsulated within the liposomes. To improve the liposomes’ tumor-targeting selectivity, the resultant liposomes were then further camouflaged with a macrophage (Mϕ) cell membrane. The liposomal formulation, called nano-Pt/VP@MLipo, was characterized and shown to have a spherical morphology, a size of around 120 nm, and an efficient integration of Mϕ cell membrane components. The goal of biomimetic membrane camouflage is to enhance circulation stability and avoid concerns regarding accelerated blood clearance. Functional experiments demonstrated that light irradiation at 690 nm, which is dependent on VP-mediated PDT, was responsible for the release of nano-Pt from nano-Pt/VP@MLipo. Furthermore, H_2_O_2_ breakdown is catalyzed by nano-Pt to produce oxygen, which increases the amount of reactive oxygen species (ROS) in tumor cells. In vitro, the liposomal formulation showed strong cytotoxicity against tumor cells and was able to significantly penetrate the agarose matrix and 4T1 tumor spheroids. Using an orthotopic 4T1 breast tumor mouse model, in vivo tests demonstrated impressive anticancer benefits including the suppression of lung metastasis, extension of survival, and inhibition of tumor development. Owing to the remarkable tolerance, liposomal delivery technology is a good option for additional research and development in cancer therapy.^27^

Phototherapy has several benefits; however, it also has drawbacks including immune recognition, blood clearance, and inadequate targeting. Researchers have developed a variety of nanocarriers, such as lipid-hybrid cell-derived biomimetic functional materials, to overcome these difficulties. These materials demonstrate promise in efficiently delivering photosensitizers to tumor tissues for enhanced therapeutic effects by fusing the immune-evading properties of tumor cell membranes with the drug-carrying capacity of liposomes.^23^ This technique improves the precision of phototherapy, leading to a more effective delivery of photosensitizers to tumor tissues and cells. This demonstrates the crucial role of lipid-hybrid cell-derived biomimetic functional materials in evading immune identification and actively targeting tumor tissues. Additionally, the possible synergistic effects of PTT and PDT were investigated, providing a thorough and promising method for the development of novel therapeutic techniques for the treatment of cancer.

## How Have Optically Active Biomimetic Imaging Agents Transformed Medical Imaging?

Optically active biomimetic imaging agents encompass a wide range of nanomaterials that engulf biomimetic structures, including both inorganic and organic ones. Gold nanoparticles (GNPs) are unique inorganic nanomaterials because of their varied forms, special properties, and ability to help with targeted drug administration. The remarkable optical properties of carbon-based nanomaterials (CBNs) such as graphene, fullerenes, and carbon nanotubes make them attractive candidates for imaging and diagnostic applications. GNPs come in a variety of sizes and forms, such as core–shell nanostructures and nanocages, and have potential uses in the encapsulation and release of drugs, especially in targeted tissues like tumors.^28^ Porous silicon NPs, which are known for their low toxicity and suitability for use in focused and minimally invasive therapies, constitute the next class of inorganic nanomaterials. Porous silicon NPs are attractive because they can be fully broken down into nontoxic orthosilicic acid.^29^ They can also be modified to release drugs into cancer cells, because of their large active surface areas. Notwithstanding the encouraging characteristics of porous silicon nanoparticles, further research is required to create intelligent, multifunctional, porous silicon nanoparticle nanocarriers and conduct thorough in vivo performance assessments.

Within the field of inorganic nanomaterials, quantum dots and lanthanide-doped upconversion nanoparticles are notable for their distinct optical characteristics. UCNPs can transform low-energy near-infrared photons into high-energy emissions, which have benefits including minimum background autofluorescence and photostability.^30,31^ The benefits of quantum dots and semiconductor nanocrystals with dimension-dominant optical properties include broad wavelength-tunable emissions and resistance to photobleaching. Notwithstanding their potential, there are important questions regarding QD toxicity that need to be investigated further and answered before they are used in clinical practice. When the focus is on organic nanomaterials, aggregation-induced emission fluorogens exhibit strong fluorescence when aggregated, and modest emission occurs at the molecular level. Aggregation-induced emission fluorogens are adaptable elements that are used in theranostic platforms, PDT, and photoacoustic (PA) imaging. Furthermore, the potential of organic semiconducting agents in near-infrared imaging was examined.^32^ These agents include semiconducting polymer nanoparticles and semiconductor molecular nanoparticles. These agents have tunable optical characteristics and high absorption coefficients but also have drawbacks, such as sluggish clearance from the body and accumulation.

**Figure 3:**
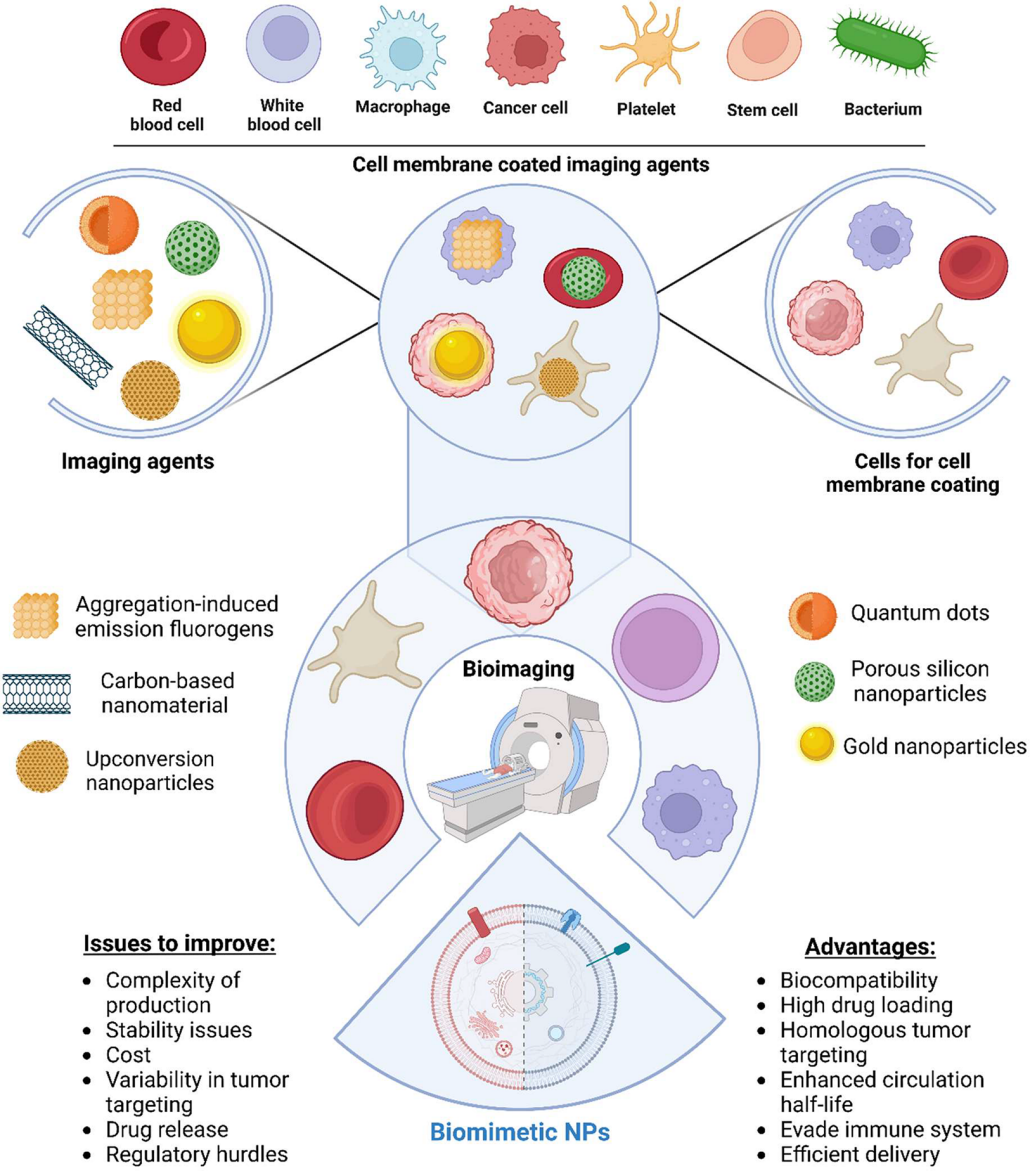
A schematic representation of the development of biomimetic NPs for bioimaging applications shows the process of utilizing various imaging agents, such as aggregation-induced emission fluorogens, carbon-based nanomaterials, upconversion nanoparticles, quantum dots, porous silicon nanoparticles, and gold nanoparticles, for coating cell membranes derived from cells (red blood cells, white blood cells, macrophages, cancer cells, platelets, stem cells, and bacteria) specifically prepared for this purpose, ultimately leading to enhanced biocompatibility and functionality in bioimaging techniques. This figure also highlights the main issues to improve biomimetic nanoparticle development, such as the complexity of production and stability issues as well as the key advantages they offer, including biocompatibility and homologous tumor targeting. This integration aims to enhance bioimaging for diagnostics and improve drug delivery systems for medical applications. Designed by Biorender.

Incorporating these imaging agent carriers into biomimetic systems provides precise delivery to the targeted site and allows the imaging of cancerous tissues.^33^ This emphasizes the significance of altering nanoparticles with cancer-targeting compounds to improve their selectivity and sensitivity, acknowledging their high extinction coefficients, fluorescence intensity, and biocompatibility. However, this underscores the need for thorough in vivo investigations to evaluate the safety and efficacy of these innovative imaging agents prior to clinical implementation.^34^ Overall, this highlights the special qualities, uses, and difficulties that require further research (Figure 3). Interestingly, biomimetic membrane nanoparticles have been studied to tackle these limitations. These are categorized based on the membrane being implemented, including RBC membrane-coated nanoparticles, immune cell membranes based on neutrophils, NK cells, T cells and macrophages, platelet cell membranes, and cancer cell and exosome membrane-based cells. First, the RBC membrane possesses the CD47 marker on its surface, which interacts with the signal regulatory protein-α (SIRPα) that gives the signal of “do not eat me”. This characteristic of RBC evades immune clearance and prolongs circulation half-life in the bloodstream. RBCs lack major histocompatibility complex (MHC) molecules, further reducing their immunogenicity. Immune cell membrane-based biomimetic nanoparticles have been used to evade immune responses. Alternatively, macrophage membrane-based biomimetic nanoparticles are widely used to express integrins (e.g., α4) on the surface of their membranes, enabling binding to vascular cell adhesion molecule-1 (VCAM-1) on the surface of cancer cells. Thus, macrophages aid in tumor targeting. Otherwise, the neutrophil membrane aids in identifying circulating tumor cells and contributes to the elimination of tumor cells. NK cell membranes are inherently cytotoxic to tumor cells, thereby stimulating the antitumor immune response. Remarkably, the platelet membrane expresses CD47 proteins on its surface, similar to the RBC membrane, thus enhancing its circulation half-life by evading the immune response. Finally, cancer cell membranes inherently possess the ability to surface markers and adhesion molecules related to the tumor and its metastasis, leading to homotypic binding to cancer cells and their interaction, enabling tumor targeting, therapy, and imaging. The exosome membrane also possesses CD47 on its surface, providing immune evasion capabilities and aiding in specific organ targeting. Thus, diverse cell membranes provide distinct characteristics, empowering the targeting ability and a unique characteristic to treat tumorous cells.

The primary challenge involved in biomimetic systems is the complexity of the membrane extraction process, which can result in significant variability from batch to batch. Additionally, the production costs and assessment of the physiological stability pose challenges for the fabrication of biomimetic nanomedicines. Their synthesis relies on the isolation and extraction of cells and their membranes. For the extraction of RBCs, platelets, and WBCs, centrifugation was employed, where the individual cells were isolated by adding fraction isolation reagents. For the collection of immune cells, including macrophages, NK cells, and neutrophils, bone marrow was collected from animals and subjected to density-gradient centrifugation. Tumor cells can be harvested from cell cultures or extracted from cancer mouse models. After cell isolation, cell membranes can be extracted by lysing the cells, removing intracellular components, and collecting the cell membranes. RBCs and platelets, which lack nuclei, can be collected by lysing the cells in hypnotic solutions and mixing with Tris solution, followed by centrifugation to remove the intracellular components. Nuclei containing cells are lysed using a hypnotic solution or mechanical destruction. Cell membranes were isolated by discontinuous sucrose gradient centrifugation and isotonic buffer washing to remove the intracellular components. The nanoparticles were cloaked within the membrane vesicles by incubation, sonication, or extrusion. The drug molecules were loaded into the nanocore by hypnotic dialysis, endocytosis, extrusion, or membrane binding. Owing to the complexity involved in their design and synthesis, biomimetic systems may face hurdles for regulatory approval, leading to clinical translation. Developing bioresponsive drug delivery systems from biomimetic systems is critical, because it is difficult to mimic the responsiveness to the environment and release the drug. The reproducibility of these systems cannot be guaranteed by genetic engineering, as it is challenging to retain the same level of membrane protein quantity. The long-term effects of genetically or chemically engineered biomimetic systems in the physiological environment need to be thoroughly assessed. It is clear that biomimetic nanoparticles enable targeted delivery with enhanced biocompatibility, long circulation time, tissue homing characteristics, the ability to cross biological barriers, and multifunctionality, including targeting, imaging, and treatment. However, membrane-based biomimetic nanomedicines encounter specific hurdles. For instance, although RBC membranes can enhance circulation time, they are relatively weak for targeted delivery. Additionally, cancer cell membranes can provoke an immune response while entering specific tissues.

## How Are Optically Active Biomimetic Therapeutic Agents Redefining Approaches to Treatment?

Significant progress has been made in the realm of biomimetic therapeutic drugs for cancer treatment, especially in relation to PDT and PTT. Numerous optically active substances have been developed to improve the effectiveness of optically active molecules. The use of photosensitizers in conjunction with NPs for PDT has gained popularity, because of several benefits. Inorganic and organic nanostructured PSs, including gold nanoparticles, metallic oxides, carbon-based materials, mesoporous silica, polymeric micelles, and upconversion nanoparticles (UCNPs), have been developed for image-guided PDT therapies.^32,35^ The emergence of aggregation-induced emission (AIE) PSs has been a notable development. AIE-based PSs show promise for image-guided PDT because they reduce nonradiative energy consumption and increase the signal intensity and ROS production in the aggregate state. TPETCAQ, a PS with AIE properties encased in a DSPE-PEG-MAL matrix to create TPETCAQ NPs, is an example.^36^ Strong fluorescence emission, elevated ROS generation, and superior PDT efficacy for tumor treatment were displayed by these NPs.

One example of success is UCNPs, which have distinct characteristics such as low toxicity, resistance to photobleaching, and the capacity to convert near-infrared emission to visible light. Long-lived red emissions from UCNPs coincide with some PS absorption, offering a viable avenue for PDT energy transfer under near-infrared radiation. Intensely red-emitting Na0.52YbF3.52:Er UCNPs have been developed for tumor PDT and multimodal imaging. Under NIR irradiation, these UCNPs showed excellent efficacy for ^1^O_2_ generation and cancer cell death. Surface modification with DSPE-PEG improves its usability in vivo.^37^ Graphene oxide (GO) nanocarriers have been used to overcome the shortcomings of conventional PDT PSs in vivo, which include poor solubility and insufficient selectivity. Several PSs have been developed for PDT and tumor imaging for loading onto the surface of GO.^38,39^ PDT efficacy and targeting are enhanced by functionalization with tumor-specific compounds, including peptides, ligands, and antibodies. For example, in animal models, a PS-loaded GO nanocomplex coupled with a tumor-selective HK peptide showed specific absorption in tumors and greatly reduced lung metastasis and tumor recurrence.^40^

**Figure 4:**
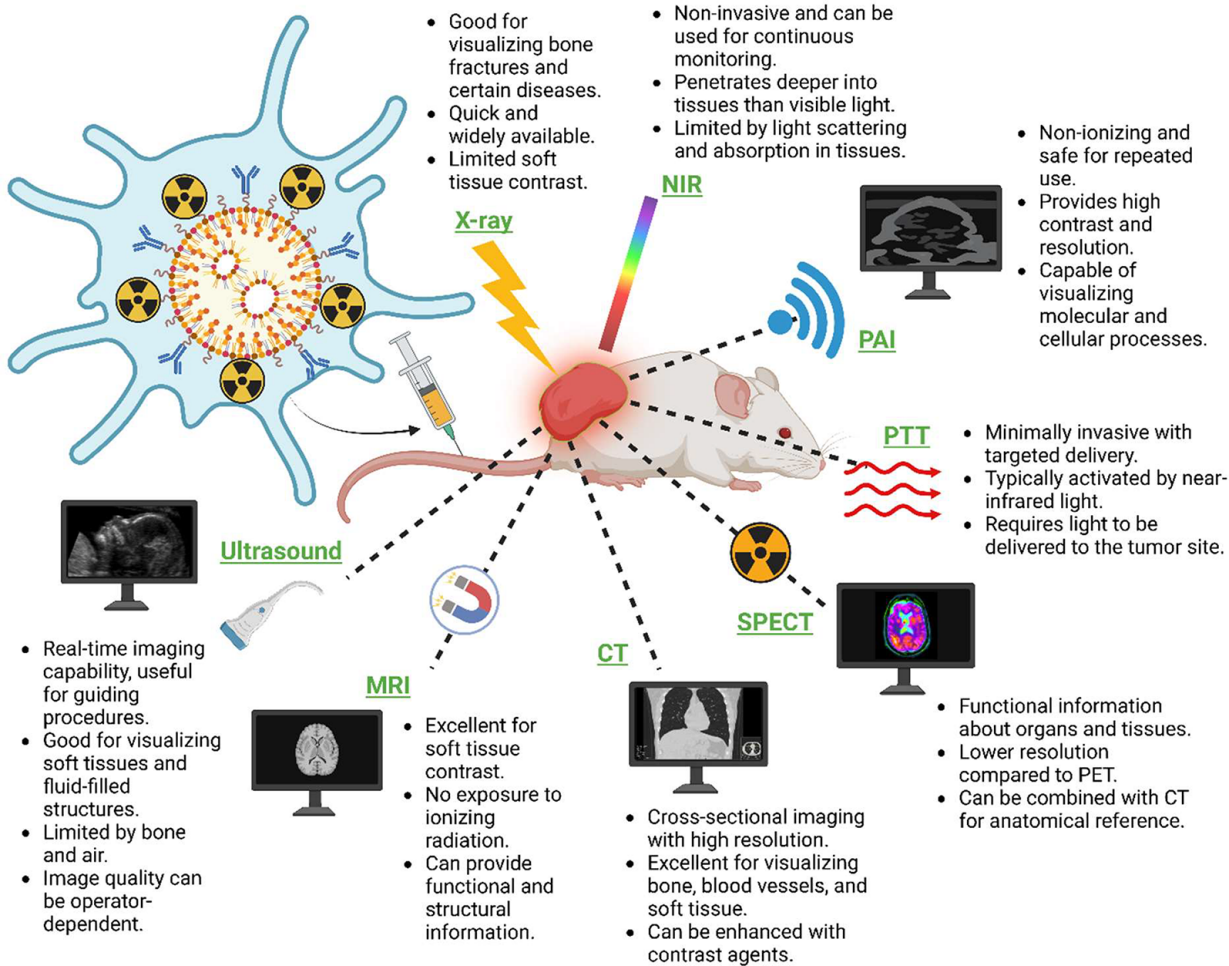
Optically active biomimetic therapeutic agents for multimodal diagnostic imaging and therapeutic approaches in nanomedicine. This diagram shows the synergistic use of biomimetic nanoparticles (NPs) with a variety of imaging and treatment techniques. This illustrates how biomimetic NPs can enhance the capabilities of X-rays, NIR, PAI, PTT, SPECT, CT, MRI, and ultrasound. Each technique has advantages, such as the nonionizing nature of NIR, real-time imaging capacity of ultrasound, and excellent soft tissue contrast provided by MRI. The figure also shows how these imaging modalities can be used in conjunction with biomimetic nanoparticles to improve diagnosis and treatment, particularly in oncology. Designed by Biorender.

The application of photothermal transduction agents (PTAs) in PTT has attracted interest. These nano PTAs come in two varieties: organic (such as semiconducting polymer NPs, nanomicelle-encapsulated NIR dyes, and porphysomes) and inorganic (such as noble metals, metal chalcogenides, carbon-based materials, and 2D materials). They have benefits such as strong NIR absorption, high photothermal conversion efficiency, and good accumulation in tumors. Two notable examples are nanodiamonds (NDDs) and stoichiometric semiconductor metal sulfide nanocrystals (e.g., Ag_2_S and CuS). The Ag_2_S nanodots demonstrated excellent circulation, tumor accumulation, and size-dependent temperature increase for successful PTT.^41^ Strong LSPRs in the NIR region were demonstrated by PEGylated Cu_2_–nSe NPs, and PTT showed that they were effective for tumor treatment (Figure 4A). Under NIR laser illumination, folic acid conjugated NDD nanoclusters demonstrated the selective ablation of tumor cells, suggesting their potential as effective agents for tumor therapy.

In summary, a variety of techniques, such as the use of NPs, AIE PSs, UCNPs, GO-based nanocomplexes, and other nano PTAs, have been employed in the production of optically active biomimetic theranostic agents for cancer treatment, indicating the versatility and advancement of the field. These developments could lead to increased accuracy and effectiveness of cancer therapy. The development of theranostic agents that originate from both organic and inorganic sources has focused on photoactive materials to circumvent the drawbacks of traditional chemotherapy in the treatment of cancer. With the combination of metallic NPs, carbon-based, noncarbon-based, and organic/inorganic nanohybrids, these nanohybrids have the potential to provide minimally invasive, synergistic therapy.^42,43^ Cell membrane-modified Fe_2_O_3_ nanoclusters implanted in polypyrrole (CM-LFPP) have been used in a biomimetic manner for photothermal therapy, guided by dual-modal imaging of prostate cancer and photoacoustic/magnetic resonance. The second near-infrared window (NIR-II) is where CM-LFPP shows significant absorption, which allows for a high photothermal conversion efficiency and superior photoacoustic imaging capabilities. With active tumor targeting made possible by lipid encapsulation and biomimetic cell membrane modification, CM-LFPP provides a high signal-to-background ratio for NIR-II photoacoustic imaging. Furthermore, CM-LFPP shows promise as a viable theranostic agent for the treatment of prostate cancer by demonstrating biocompatibility and enabling low-dose photothermal therapy for tumors. Evaluations of CM-LFPP both in vitro and in vivo demonstrated its concentration-dependent photothermal behavior, high efficiency of photothermal conversion, and dual-modal imaging capabilities, making it a flexible tool for diagnosis and treatment. The extended retention time of CM-LFPP in the tumor regions is indicative of its active tumor-targeting capability, offering a major benefit for precise detection (Figure 4B). Furthermore, CM-LFPP exhibits promising properties as a multifunctional theranostic agent with high sensitivity and specificity for prostate cancer, because it functions as an efficient contrast agent for photoacoustic and magnetic resonance imaging.^44^

In conclusion, the integration of optically active biomimetic therapeutic agents, including photosensitizers and photothermal transduction agents, with advanced nanotechnology has revolutionized the field of cancer treatment. These innovative strategies not only enhance the specificity and efficiency of tumor targeting but also minimize side effects and improve patient outcomes. Synergy between optically active molecules and nanocarriers has improved the development of highly effective and minimally invasive therapeutic modalities. As research in this area continues to advance, it holds promise for providing more personalized and precise treatment options for cancer patients. The future of cancer therapy lies in further exploration and optimization of these biomimetic approaches, potentially leading to breakthroughs in the treatment of various malignancies. Ongoing advancements in the field underscore the importance of interdisciplinary collaboration among scientists, engineers, and clinicians to harness the full potential of optically active biomimetic therapeutic agents in redefining approaches to cancer treatment.

## How is Biomimetization Transforming the Field of Cancer Nanomedicine?

Cancer remains a grave global threat, with cancer-related fatalities surpassing those caused by cardiovascular disease. Approximately one-fifth of all human deaths is attributed to cancer. While conventional cancer treatments, such as surgery, chemotherapy, and radiotherapy, are clinically approved, they often lack efficacy due to incomplete tumor removal and the persistence of circulating tumor cells. Therefore, there is a pressing need for therapeutic approaches that are convenient, highly specific, and efficient, with minimal side effects.^45^ Localized therapies offer promise in cancer treatment because of their precise targeting, which alters drug distribution in vivo compared to intravenous injections. However, this method requires frequent chemotherapy injections, posing challenges for patients such as pain and potential complications. To address this issue, researchers are shifting their focus to drug-delivery platforms that enable sustained and controlled drug release throughout the treatment cycle.^46^ Numerous polymer-based drug delivery systems (DDSs) have been explored to directly target tumors and release drugs as polymers naturally degrade. However, controlling the release rate has proven to be challenging. Uncontrolled release can lead to ineffective therapy owing to inadequate drug concentration, and worse, it can increase the risk of cancer cells developing resistance. Therefore, the development of a controlled drug delivery system is imperative to enhance cancer treatment outcomes while minimizing patient discomfort and complications.^47^

Notably, researchers have explored phototherapy as an alternative and safe treatment approach in which Nd^3+^-sensitized upconversion NPs excited with an 808 nm laser offer high luminescence intensity, increased penetration depth, and reduced tissue overheating. This 808 nm NIR light can also serve as the excitation source for various photosensitive agents, creating a dual-excitation effect that enhances therapeutic outcomes. Efforts have been made to combine the effects of PTT and PDT into a single anticancer system to maximize therapeutic efficacy.^48^ Photothermally active NPs have gained significant importance in cancer therapy because of their unique ability to convert light energy into heat, which leads to localized hyperthermia and subsequent tumor destruction. In targeted therapy, photothermally active NPs can be designed to specifically target cancer cells. Functionalization of NPs with ligands or antibodies enables precise targeting and minimizes damage to healthy cells. Hao et al. aimed to create versatile poly(lactic-*co*-glycolic) acid (PLGA) NPs, decorated with angiopep-2, to deliver both indocyanine green (ICG) for NIR imaging and phototherapy, and docetaxel (DTX) for chemotherapy to the brain. This design enables the use of combined chemophototherapy for glioma.^49^ These NPs can serve as contrast agents for various imaging techniques, including photoacoustic and thermal imaging, and they provide real-time feedback during treatment. Zhang et al. explored recent advancements in PDT, a treatment combining light and photosensitizers to generate ROS for cellular damage in cancer and infectious diseases. The focus included new photosensitizer designs, genetic engineering of biological photosensitizers, and the use of PDT-induced inflammation for therapeutic delivery in deep tumor tissues. PDT combined with immunotherapies shows promise in cancer treatment and has been explored to overcome antimicrobial resistance in bacterial infections.^50^

**Figure 5:**
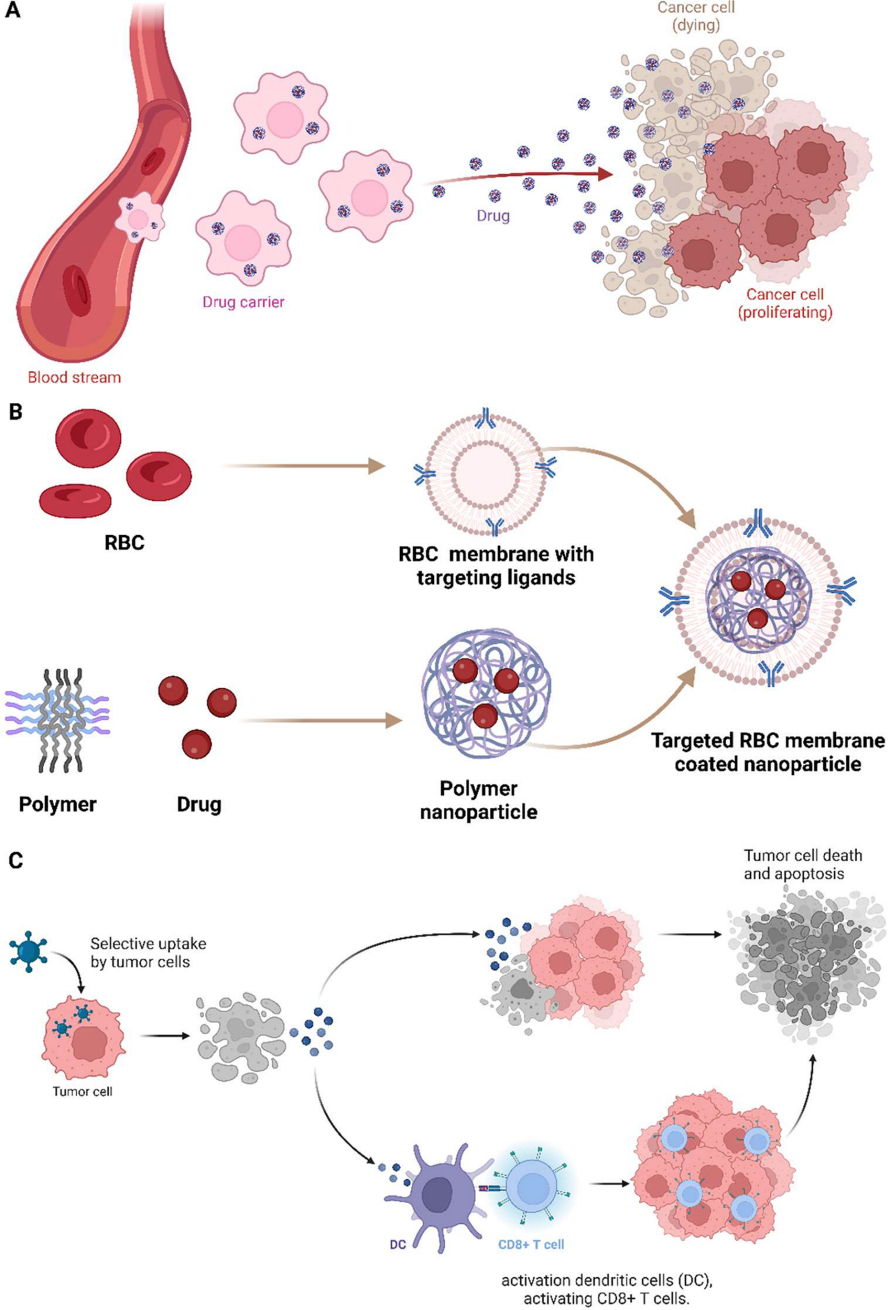
Biomimetics of cancer nanomedicine. (A) Drug carriers in the bloodstream release drugs near proliferating cancer cells, with some cancer cells depicted as dying owing to drug effects. (B) The process of creating targeted RBC membrane-coated nanoparticles by cloaking polymer nanoparticles with RBC membranes that have targeting ligands, which are then loaded with drugs. (C) Biomimetic immunotherapy via the selective uptake of these targeted nanoparticles by tumor cells, leading to tumor cell death and apoptosis and the subsequent activation of dendritic cells (DC) and CD8+ T cells, which are crucial for the immune response to the tumor. Designed by Biorender.

Photothermally active NPs can be combined with other therapies, such as chemotherapy or immunotherapy, to enhance the overall treatment efficacy through synergistic effects. Chen et al. described a strategical therapeutic approach that combines NP-based PTT using ICG and Toll-like-receptor-7 agonist imiquimod (R837) coencapsulated in PLGA with anticytotoxic T-lymphocyte antigen-4 (CTLA4) checkpoint-blockade immunotherapy (Figure 5). PLGA-ICG-R837 NPs, composed of clinically approved components, enable NIR laser-triggered photothermal ablation of primary tumors.^51^

Researchers are progressively exploring inorganic matrices, including silica, gold, iron oxide, and quantum dots, in an effort to augment the potential of nanoparticles for concurrent imaging and therapeutic uses.^52^ As a result, theranostics, a single nanoparticle with both therapeutic and imaging properties, was developed. There are several benefits of using nanoparticles for both diagnosis and treatment in cancer applications, such as cancer nanotheranostics. By addition of imaging properties to nanoparticles, the distribution of treatments can be tracked in vivo and in real time, offering important insights into the mechanism of action. The emerging field of image-guided cancer nanomedicine, in conjunction with interventional oncology methods, ensures minimal systemic distribution, homogeneous targeting, and high local delivery of nanomedicine, thereby enhancing the efficacy of advanced nanomedicines. Image-guided nanomedicine delivery is important for future clinical applications. First, it allows the precise localization of nanomedicines within tumor regions, minimizing systemic toxicity. Second, it enables real-time monitoring to confirm the proper delivery of nanoparticle-based nanomedicines to the disease site (local infusion and tracking). Third, the quantity of injected nanoparticles can be quantitatively analyzed to determine postinfusion amounts (noninvasive quantification). Finally, long-term monitoring of the nanoparticle distribution in the body facilitates ongoing diagnosis and evaluation. Image-guided cancer nanomedicine integrates cutting-edge medical imaging technologies, nanoparticles, molecular entities, and innovative therapeutic agents (such as siRNA, mRNA, gene editing tools, and immune checkpoint inhibitors)^53,54^ with existing drugs and therapeutics. The progress of image-guided cancer nanomedicine in clinical applications requires collaborative efforts from multidisciplinary teams, including clinicians, basic scientists, and nanoscientists working closely together. This collaborative approach is vital for the successful advancement of image-guided cancer nanomedicine for clinical translation.^55^ Although the clinical development of theranostic agents is still in its early phases, problems, such as effective and focused guidance of therapeutic/imaging nanoparticles, continue to exist. The potency of nanoparticles may be further increased by the application of multimodal therapy approaches.^56^ Biomimetic nanoparticles are a noteworthy nanoparticle platform that has progressed from the benchtop to the therapeutic bedside.

## Future Directions and Conclusions

Several approaches can be explored to develop biomimetic nanoparticles integrated with imaging and therapeutic probes and to make them optically active and suitable for solid tumor optotheranostic applications. However, various engineering and biological barriers traffic them between the interstitium, lymphatic system, and blood circulation before reaching the tumor site. Thus, a revolutionary strategy for developing theranostic applications in the field of solid tumors involves combining nanomedicine with biomimetic cell ghost-lipid nanostructures. Combining phototherapy with nanotechnology provides a sophisticated approach to enhance drug delivery efficiency across biological membranes while utilizing the unique characteristics of photoactive substances. The targeting capabilities of this paradigm are further improved by the addition of biomimetic cell ghost-lipid nanostructures, which guarantee the targeted delivery of nanomedicine to the tumor microenvironment while minimizing adverse effects on normal tissues. By extending the circulation half-life and avoiding clearance obstacles presented by the reticuloendothelial system (RES), this biomimetic approach promotes an increased biocompatibility. A significant breakthrough in the treatment of solid tumors is the combination of immunotherapy and phototherapy in this all-encompassing framework, which offers diverse therapeutic strategies. Combining the two modalities not only improves treatment precision but also maximizes synergistic benefits for better therapeutic outcomes. Concurrent advancements in imaging technology and photonanomedicine provide physicians with precise diagnostic instruments to help them make informed decisions about customized treatment strategies. In addition, biomimetic cells are an important element beyond the field of nanomedicine, serving as immunomodulatory agents to increase the therapeutic effect against solid malignancies. Future directions for this field of study include further investigation of the synergistic potential of biomimetic cell ghost-lipid optotheranostics. To maximize the therapeutic impact of these nanostructures through improved drug delivery and personalized therapies, researchers are working to improve their functionality and design. The potential to revolutionize cancer therapy paradigms and advance precise therapy in the clinical management of solid tumors exists with the translation of these novel techniques from the bench to bedside. Current developments in this field are likely to bring about a new era of treatment approaches, providing hope for better patient outcomes and ultimately influencing the development of cancer therapy techniques. Recent research in the field of biomimetics is summarized in Table 1. Research in this field needs to continue to address a few potential questions, including studying the mechanism by which biomimetic nanosystems enter target cells, understanding the process of targeting the site of action, and exploring how biomimetics evade host recognition.

**Table 1:** Recent Research in the Field of Biomimetics Focused on the Type of Nanomaterial, Specific Applications, and Key Functional Improvements and References to Seminal Studies

biomimetic carrier	PTT and/or PDTPDT	tumor	inference	ref
leukocyte/platelet hybrid membrane	IR780	4T1 cells inoculated mice	excellent targeting ability and very high in vitro and in vivo PTT/PDT performances	(57)
erythrocyte membrane	zinc phthalocyanine, ICGICG	4T1 tumor-bearing nude mice	exhibited excellent phototherapeutic efficacy in xenograft nude mouse models, thereby achieving complete tumor ablation in a single treatment cycle	(58)
cancer cell membrane	ICG, Nrf2-siRNANrf2-siRNA	SCC-25 cells into the groins of mice	showed synergistic effects of PTT and Nrf2-siRNA amplified PDT by blocking the activation of Nrf2-based antioxidant pathway	(59)
cancer cell membrane	porphine	SKOV3 cells implanted mice	demonstrated promising efficacy against ovarian cancer in vitro and in vivo, offering a potential therapeutic strategy with enhanced PTT and PDT	(60)
mesenchymal stem cell	chlorin e6 (Ce6)-conjugated polydopamine	B16-F10 cells bearing mice	induced potent phototoxicity to eliminate both the tumor cells	(61)
cancer cell macrophage membrane camouflaged persistent luminescent nanoparticles	Zn_1.25_Ga_1.5_Ge_0.25_O_4_:Cr^3+^,Yb^3+^,Er^3+^ (ZGGO) nanoparticles were coated with mesoporous silica (ZGGO@SiO_2_)	CT26-tumor-bearing mice	not only diagnosed and real-time traced colorectal cancer with long persistent luminescence imaging but also produced precisely combined chemotherapy and PTT	(62)
gene-engineered exosomes-thermosensitive liposomes hybrid nanovesicles	ICG	CT26 xegographted mice	ICG and R837 coencapsulated hGLV (I/R@hGLV) with 808 nm laser irradiation successfully eliminated the homologous CT26 tumors xenografted in mice through combination of PTT and immunotherapy	(63)
RBC membrane camouflaged semiconducting polymer nanoparticles	semiconducting polymer	4T1 tumor-bearing mice	prolonged systematic circulation time, less reticuloendothelial system uptake and reduced immune-recognition, hence improving tumor accumulation after intravenous injection, which provides strong photoacoustic signals and exerts excellent photothermal therapeutic effects	(64)
enveloped mesoporous silica nanoparticles with RBC membrane ghosts	ICG	A549 tumor-bearing mice	MSN@RBC nanoparticles displayed a size-dependent behavior, where larger particle sizes were more easily captured by the organs	(65)
PLGA nanoparticles coated with an A549 cancer cell membrane	ICG	mice bearing A549 tumor xenografts	hold great potential for multimodality imaging-guided photothermal tumor ablation	(66)
RBC	aloe-emodin	HSC-3 tumor cells xenografted Balb/c nude mice	the functional combination of PDT-induced apoptosis and nonapoptotic ferroptosis enhances therapeutic effects, but AE’s maximum absorption in the blue region limits its use to superficial diseases like skin cancer, oral cavity, and eye diseases	(67)
neutrophil membranes hypocrellin B nanoparticles	hypocrellin B	HCC tumor bearing mice	promoted ROS production and mitochondrial dysfunction via inhibit JUNB expression, not only effective as therapeutic drug for HCC, but also for highly efficient NIR FL imaging	(68)
erythrocyte membrane	gold nanorods	PANC-1 or BXPC-3 tumor-bearing nude mice	efficient photothermal/gene combination therapy with fluorescence and MR imaging capabilities with gold nanorods and plectin1	(69)
4T1 membrane-coated nanozyme	Ce6	4T1 tumor-bearing mice	developed a biomimetic smart carbon nanozyme (CCM) integrating an O_2_ generator and dual-glutathione (GSH) depleting agent, demonstrating enhanced PDT with real-time imaging and diagnostics capabilities	(70)

The field of biomimetic cell ghost-lipid optotheranostics is set to explore the synergistic opportunities offered by these advanced nanostructures. By refining their design and enhancing their functionality, researchers aim to fully harness the therapeutic potential of these systems. These efforts are directed toward optimizing drug delivery mechanisms, which are crucial for the effective treatment of various cancers. This optimization includes improving the targeting accuracy of these nanostructures to tumor sites, reducing off-target effects, and enhancing the bioavailability of the therapeutic agents. Furthermore, there is a concerted push toward personalization of therapy, which involves tailoring treatment modalities to the specific genetic and phenotypic characteristics of an individual’s tumor. This approach not only improves the efficacy of the treatment but also significantly reduces the potential for adverse side effects. The transition from benchtop research to clinical applications appears to be increasingly feasible and holds promise for revolutionizing the treatment of solid tumors. Interdisciplinary collaboration is a key driver of innovation in this field. By combining insights from materials science, molecular biology, pharmacology, and clinical oncology, researchers are developing treatment strategies that are not only effective but also aligned with the main goal of enhancing the patient quality of life. These strategies are moving toward creating treatment options that are less invasive, more effective, and capable of providing significant patient-centric benefits. As the field progresses, ongoing research and development promote major discoveries in cancer therapy. These developments promise to extend the life expectancy of patients and improve their overall quality of life. The synergy between advanced biomimetic materials and cutting-edge cancer treatment technologies is setting the stage for a new era in cancer therapy, in which the focus is on curing the disease while simultaneously ensuring the highest possible quality of life for patients.
